# The Kaleidoscope of Polyautoimmunity: An Odyssey of Diagnostic Dilemmas

**DOI:** 10.7759/cureus.57799

**Published:** 2024-04-08

**Authors:** Priyanshu Tripathi, Divendu Bhushan, Ayan Banerjee, Mala Mahto

**Affiliations:** 1 Biochemistry, All India Institute of Medical Sciences, Patna, Patna, IND; 2 General Medicine, All India Institute of Medical Sciences, Patna, Patna, IND

**Keywords:** rheumatoid arthritis, sle, mixed connective tissue disease, overlap syndrome, polyautoimmunity

## Abstract

Diagnostic accuracy is of the utmost importance, both in the clinical setting and for research purposes. Mixed connective tissue disease (MCTD), rheumatoid arthritis (RA), Sjogren's syndrome (SS), and overlap syndrome (OS) frequently exhibit symptoms that mimic those of other conditions. Unfortunately, there is no singular definitive test for diagnosing these connective tissue diseases (CTDs), necessitating the reliance on expert opinions. Further complicating the matter, these diseases have overlapping clinical and serological features, and some individuals with one autoimmune disease may develop additional autoimmune disorders, either concurrently or at a later stage of their ailment. Autoimmune diseases (ADs) may manifest as a single AD or, concurrently with other ADs, a condition named polyautoimmunity (polyA). Polyautoimmunity refers to the presence of numerous autoimmune disorders in a single patient. Multiple autoimmune syndrome (MAS) is a condition that occurs when three or more autoimmune illnesses coexist. Moreover, the coexistence of two or more ADs with classification criteria is named “overt polyA," whereas the presence of autoantibodies not related to the index AD, without criteria fulfillment, is termed “latent polyA." Furthermore, both conditions can exist simultaneously within an individual patient. This case report's findings underscore that patients exhibiting both latent and overt polyautoimmunity tend to group, exhibiting distinct clinical and immunological characteristics. Additionally, CTDs not only have overlapping features amongst their various subclasses but also tend to mimic other conditions due to an underlying chronic inflammatory state. This case study also attempts to highlight the diagnostic dilemmas faced in such situations.

## Introduction

Autoimmunity refers to the immunological reaction where the immune cells are targeted against self-antigens, resulting in autoimmune diseases [1]. These disorders have several clinical signs and symptoms, pathophysiological mechanisms, and genetic factors in common, leading to the existence of an autoimmune tautology, indicating that they share many common pathways of causation [1].

The mosaic of autoimmunity exhibits multi-factorial origin, and the diversity of autoimmune disease (AD) expression is frequently identified using classification criteria. They have sub-phenotypic similarities, such as signs and symptoms, non-specific autoantibodies, and other immunological alterations that are subject to categorization issues [2]. One of the strongest arguments supporting autoimmune tautology is polyautoimmunity, defined as the presence of two or more ADs in a single patient. The primary differentiation between overlapping syndromes and polyautoimmunity lies in their clinical manifestations. While polyautoimmunity indicates the presence of partial signs and symptoms from multiple autoimmune diseases (ADs), overlapping syndromes (OS) involve the concurrent existence of well-established clinical manifestations fulfilling confirmed classification criteria for distinct autoimmune disorders. Multiple autoimmune syndrome (MAS) is defined as the coexistence of three or more autoimmune disorders. Polyautoimmunity holds significance in the ongoing conversation as it has the potential to impact the severity of autoimmune diseases. This aspect becomes relevant due to conflicting perspectives within the scientific community. Some researchers believe that the presence of polyautoimmunity exacerbates the manifestation of a specific AD, leading to a more severe clinical outcome, whereas others believe that it does not exert any substantial influence or may, in fact, result in a more favorable prognosis for individuals with ADs. Due to their chronic nature, these ADs place a substantial strain on both social and medical resources, while also affecting the overall quality of life [2]. The capacity to diagnose the onset of these illnesses in the pre-symptomatic phase and to forecast their progression would mark a significant advancement in primary, secondary, and tertiary prevention [1]. However, the establishment of the diagnosis of connective tissue diseases (CTDs) is as complex as the pathogenesis of polyautoimmunity [3]. We try to present the challenges faced in a clinical diagnostic laboratory while evaluating a known case of rheumatoid arthritis on methotrexate who presented with a new set of symptoms necessitating a battery of investigations to possibly establish a concrete diagnosis. Autoimmune disease testing in the form of a line immunoassay for anti-nuclear antibody detection revealed positivity to multiple autoantigens, further complicating the case.

## Case presentation

**Figure 1:**
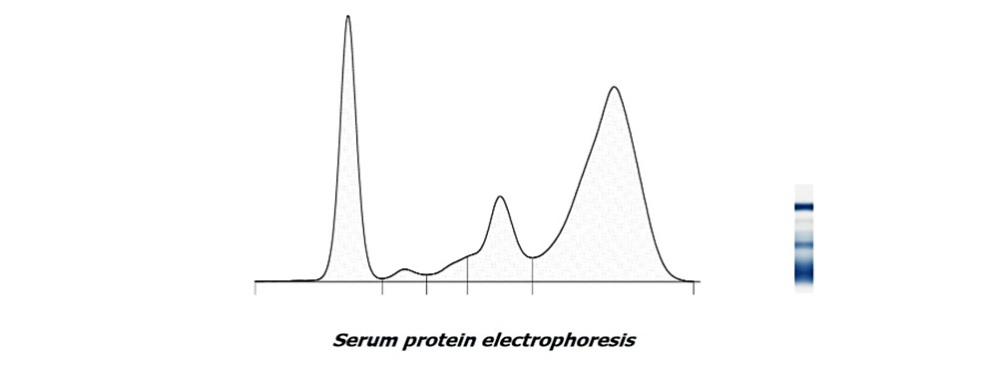
Serum protein electrophoresis The serum protein electrophoresis done by the Sebia Hydrasys 2 scan showed a sharp peak in the gamma globulin region with decreased albumin and alpha-2 globulin and increased gamma and beta globulin. (The X axis denotes the migration of plasma proteins against time, and the Y axis denotes the OD.) The algorithm for the same is governed by software, which is a proprietary product of the manufacturer of the machine. The graph corresponds to the run obtained on the gel, as shown by the different blue bands on the right side. OD: optical density

**Figure 2:**
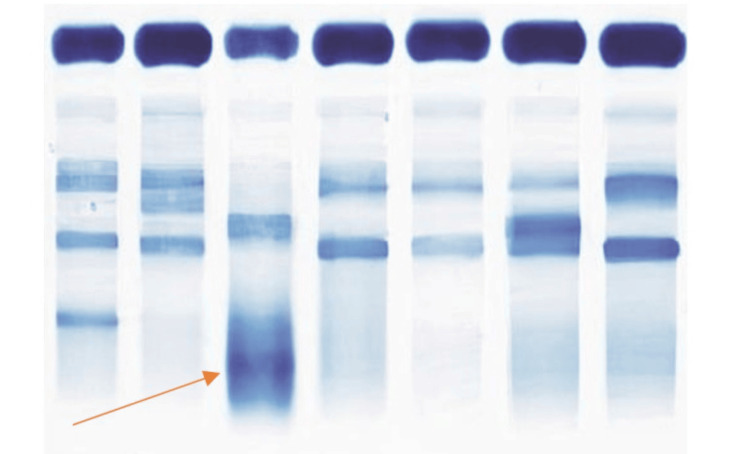
Gel run of serum protein electrophoresis (SPE) The gel film of SPE in lane 3 marked by an arrow shows a constricted band.

**Figure 3:**
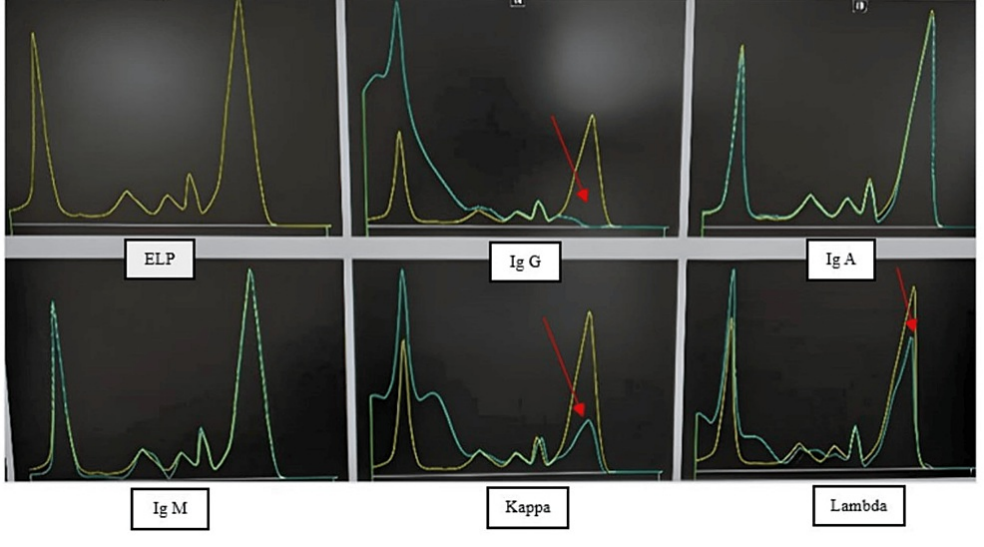
Immunotyping on Capillary 2 FP Immunotyping depicts polyclonal gammopathy with incomplete subtraction in the IgG lane. ELP depicts the electrophoresis lane; IgG depicts sera treated with anti-IgG; IgA depicts sera treated with anti-IgA; IgM depicts sera treated with anti-IgM; kappa depicts sera treated with anti-kappa; and lambda depicts sera treated with anti-lambda.

**Figure 4:**
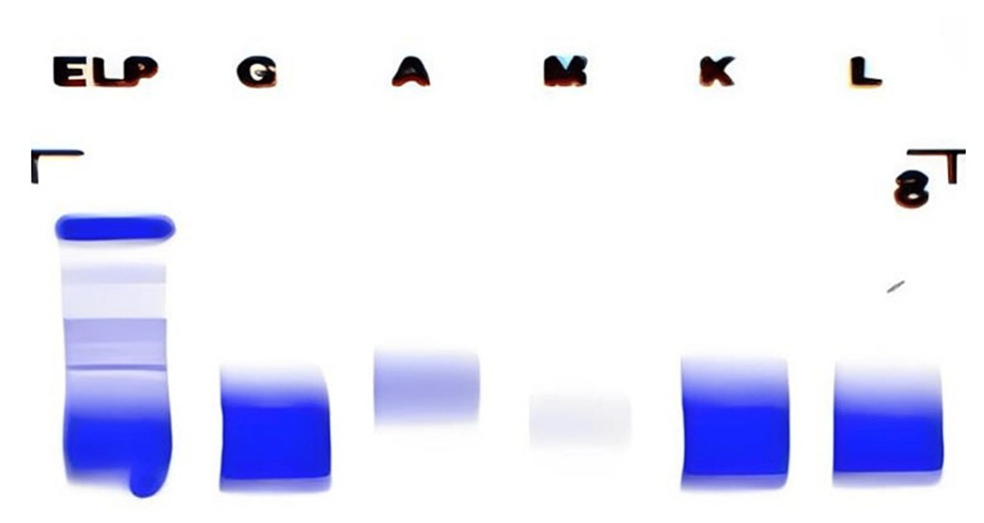
Serum protein immunofixation The serum immunofixation showed no bands with different antisera (anti-IgG, IgA, and IgM heavy chains and anti-kappa and lambda (free and bound) light chains), hence monoclonal protein was not present in the sample.

**Figure 5:**
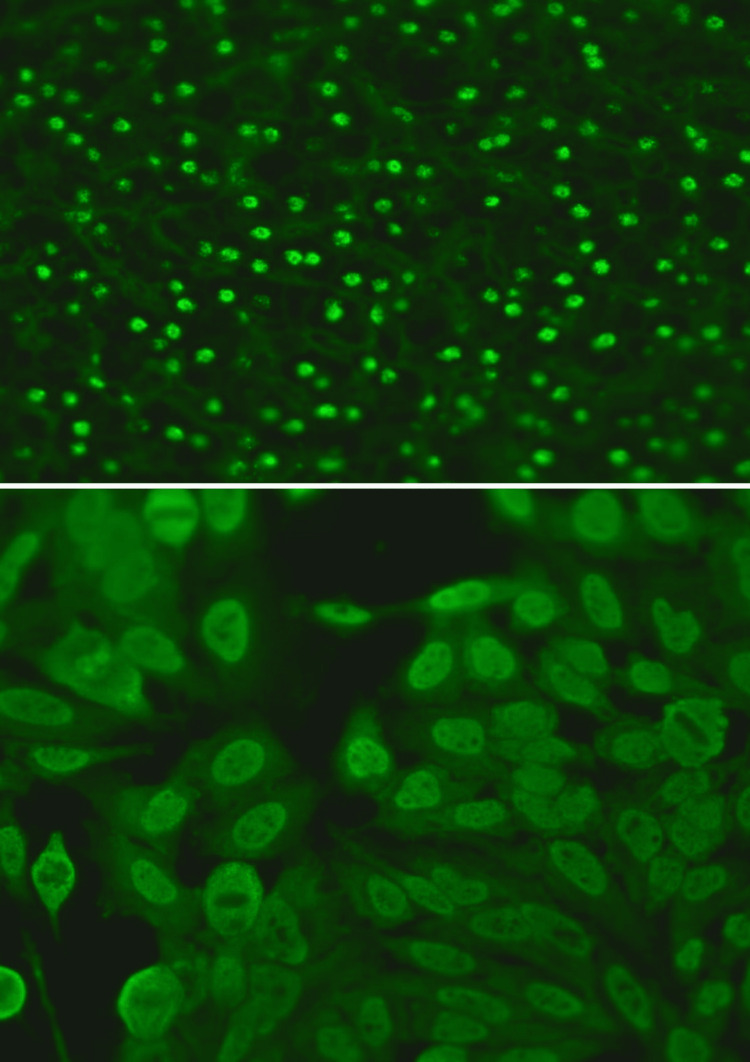
ANA screening using HEp-2 and primate liver as substrate by IIFT A speckled pattern was noted (4+ in intensity) ANA: antinuclear antibody

**Figure 6:**
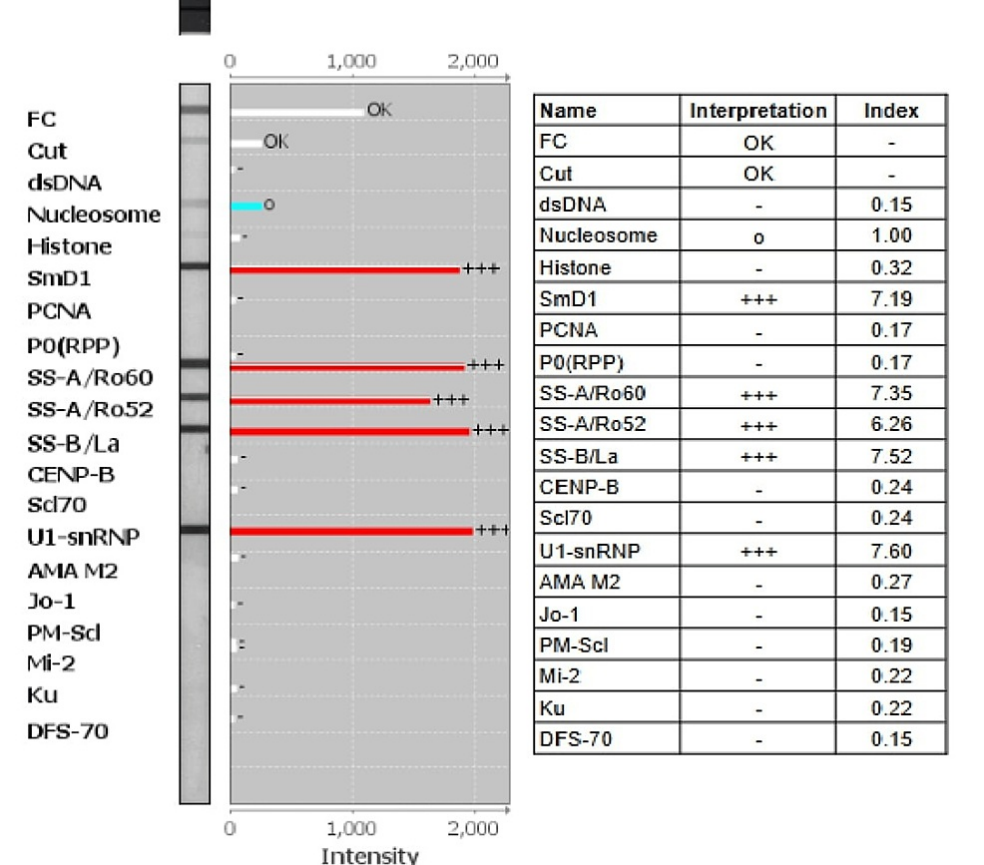
ANA profile testing by line immunoassay (LIA) LIA findings depict the presence of antibodies to multiple antigens.

A 63-year-old male presented to the medicine outpatient department (OPD) in March 2022 with complaints of intermittent fever, easy fatiguability, and dryness of the mouth (oral sicca) for the past four years, with an associated weight loss of 24 kg. For the last four months, there has been a history of breathlessness during exertion. There was an associated history of difficulty getting up from a squatting position, overhead abduction movements, and bending forward, with gradual progression leading to severe restrictions on his movements. The patient had a known case of diabetes mellitus and hypothyroidism on treatment. He was known to have rheumatoid arthritis on methotrexate. The patient was a farmer by profession and a resident of Bihar, a northern Indian state. He belonged to a middle-class background with no significant history of exposure to occupational hazards. However, he had a history of smoking cigarettes for the last 40 years (four to five a day). For four years, he was also known to have onychomycosis of the left hand. As baseline hematological investigations revealed pancytopenia, a serum protein electrophoresis (SPE) was advised. SPE (Sebia Hydrasys 2 scan) findings reported the presence of an M band amidst polyclonal gammopathy (Figures 1, 2). However, when the same sample was subjected to immunotyping (Capillary 2FP, Sebia), immunofixation electrophoresis (Hydrasys 2, Sebia) (Figures 3, 4), and serum-free light chains (SFLCs) (Atellica NEPH630), the results favored a polyclonal pattern. Antinuclear antibody (ANA) testing by indirect immunofluorescence microscopy (IIFT Euroimmun Mosaic Hep-20-10/Liver) showed a highly positive (4+) speckled and cytoplasmic pattern (Figure 5). Line immunoassay (LIA) (HumaBlot 44 FA) for the ANA profile was reported to be strongly positive for SmD1, SSA/Ro 52, SSA/Ro 60, SSB/La, and U1snRNP (+++). Antibodies against nucleosomes by LIA were equivocal (Figure 6). Rheumatoid factor (Beckman Coulter AU680) and anti-cyclic citrullinated peptide (anti-CCP) were negative (Siemens, ADVIA CENTAUR XPT). Bone marrow aspiration revealed 9% plasma cells. Contrast-enhanced computed tomography (CECT) of the thorax and abdomen revealed findings suggestive of early non-specific interstitial pneumonitis (NSIP). Hepatomegaly was noted with bilaterally enlarged inguinal lymph nodes. Interleukin 6 (IL-6) was within the normal range. The patient did not have any hypercalcemia, renal dysfunction, anemia, and bone (CRAB) lesions except for anemia with hemoglobin at 9.2 gm/dl. Pancytopenia was observed on a hematological examination. Total protein was elevated to 9.97 gm/dl, albumin decreased to 1.85 gm/dl, and the A:G ratio was reversed to 0.23. Serum IgG by nephelometry was elevated at 60.3 gm/l. The free kappa/lambda ratio was normal at 1.45, though both kappa and lambda light chains were raised to 344 mg/l and 237 mg/l by nephelometry. Table 1 displays detailed laboratory investigations.

**Table 1:** Laboratory parameters during the first visit and follow-up visit MCV: mean corpuscular volume; MCH: mean corpuscular hemoglobin; MCHC: mean corpuscular hemoglobin concentration; AST: aspartate aminotransferase; ALT: alanine aminotransferase; ALP: alkaline phosphatase; LDH: lactate dehydrogenase; CRP: C-reactive protein; RDW-CV: red cell distribution width-coefficient of variation; WBC: white blood cells; PCV: packed cell volume; anti CCP: anti-cyclic citrullinated peptide; RA: rheumatoid arthritis

Lab parameters				March	October
Liver function test (LFT)	Investigation	Reference range	Units	Results	Results
	Total bilirubin	0.3 - 1.2	mg/dl	0.39	0.52
	Direct bilirubin	< 0.3	mg/dl	0.12	0.12
	Indirect bilirubin	< 1	mg/dl	0.27	0.40
	ALT/SGPT	13 - 40	U/L	54.4	80.2
	AST/SGOT	< 37	U/L	155.7	277.8
	ALP	30 - 90	U/L	79.9	475.8
	Total protein	6.4 - 8.3	g/dl	7.48	5.17
	Albumin	3.4 - 4.8	g/dl	2.37	2.74
	Globulin	2 - 3.5	g/dl	5.11	2.43
	A/G ratio	1.5-2.5	g/dl	0.46	1.13
Kidney function test (KFT)	Serum urea	13 - 43	mg/dL	38.1	37.5
	Creatinine	0.7 - 1.3	mg/dl	0.58	0.36
	Uric acid	3.5 - 7.2	mg/dl	5.76	4.27
	Serum calcium	8.6 - 10	mg/dL	7.68	6.96
	Phosphorus	2.7 - 4.5	mg/dl	2.92	3.42
	Sodium	135 - 145	meq/l	123.13	119.13
	Potassium	3.5 - 5	meq/l	3.90	4.88
	Chloride	98 - 107	meq/l	96.27	87.98
Complete blood count (CBC)	Hemoglobin	13 - 17	g /dl	9.5	10.7
	WBC	4 - 10	Thousand/MicroLtr /dL	2.80	15
	Platelet count	50 - 450	Thousand/MicroLtr	87	167
	RBC count	4.5 - 5.5	Thousand/MicroLtr	3.13	3.76
	PCV	40.0 - 50.0	%	2.87	33.0
	MCV	83 - 101	fL	91.7	87.8
	MCH	27 - 32	pg	30.3	28.5
	MCHC	31.5 - 34.5	g/dL	33.1	32.4
	RDW CV	11.6 – 14	%	17.0	14.6
	Neutrophils	40 - 80	%	79.6	95.2
	Lymphocytes	20-40	%	17.5	2.7
	Monocytes	2-10	%	1.8	1.7
	Eosinophils	1-6	%	0.7	0.3
	Basophils	0-1	%	0.4	0.1
	Mean platelet volume	9.4-12.3	fL	12.2	11.5
	Platelet distribution width	10-17.9	%	15.9	14.9
CRP quantitative		0 - 5	mg/l	30.65	90.53
R Factor		< 10	IU/ML	<10.60 (previous reports showed RA of 126)	
ANTI CCP		< 20	U/ml	1.08	

Given overlapping findings, a provisional diagnosis of mixed connective tissue disorder was made. He was administered 40 mg/day of wysolone for 10 days and then advised to taper off. The patient was advised to follow up after three months. As the patient’s condition failed to improve, he was referred to a rheumatology center in June 2022, where a provisional diagnosis of late-onset systemic lupus erythematosus (SLE) with lupus nephritis was made. A bilaterally thickened common peroneal nerve involving the lower limb was noted, and a nerve conduction study revealed sensory-motor polyneuropathy. A kidney biopsy was planned but could not be carried out given the isolated raised prothrombin time (PT)/international normalized ratio (INR). Clinical manifestations of arthritis, malar rash, and photosensitivity are usually less frequently noted in late-onset SLE affecting individuals > 49 years of age. He was administered three doses of pulse methylprednisolone, followed by oral omnacortil, hydrochloroquine, and mycophenolate mofetil. The patient developed an abscess on the right hand and a fever in October 2022. There were associated complaints of increased shortness of breath on exertion. Urine output was reduced. The patient finally succumbed to his illness in November 2022.

## Discussion

Based on the clinical spectrum and antibodies associated with various phenotypes, systemic autoimmune diseases have been classified under six mutually exclusive diseases, which include systemic lupus erythematosus (SLE), systemic sclerosis (SSc), dermatomyositis (DM), polymyositis (PM), primary Sjögren syndrome (pSS), and rheumatoid arthritis (RA). These diseases are labeled as definitive connective tissue diseases (DCTD). However, some of the cases of systemic connective tissue disease do not fulfill the criteria of a DCTD or have the criteria of more than one disease. This led to the concepts of undifferentiated connective tissue disease (UCTD), mixed connective tissue disease (MCTD), and overlap syndrome (OS). UCTD refers to that group of CTD that does not have specific clinical features but possesses positive autoantibodies. However, a UCTD diagnosis should not be made until the patient remains unclassifiable for at least three years, as a significant percentage of early UCTD gradually evolves to DCTD or an OS (35%). Cases with a shorter duration should be referred to as early UCTD. It has also been reported that a DCTD may evolve into another DCTD with time. OS is diagnosed when the patient meets the classification criteria of at least two different DCTDs. It differs from MCTD, which is an evolved OS but inextricably linked with the anti-U1snRNP antibody and amalgamates the overlapping features of SLE, SSc, and idiopathic myositis [1]. MAS is described as the co-occurrence of three or more different autoimmune diseases (ADs) within a single patient.

The above-described case involves a patient who was diagnosed with rheumatoid arthritis along with other comorbidities and was being treated with methotrexate. Rheumatoid factor (RF) and anti-CCP were within the normal reference range at the time of presentation to our hospital. A suspicion of monoclonal gammopathy was raised given pancytopenia, anemia, and a raised serum total protein with a reversal of the A:G ratio. SPE revealed the presence of a sharp band amidst polyclonal gammopathy. However, further confirmatory tests like serum immunotyping and SFLC ruled out monoclonal gammopathy. Bone marrow aspiration and biopsy results ruled out multiple myeloma, as plasma cells were 9%. The possible presence of a sharp band on SPE and 9% plasma cells on bone marrow aspiration and raised serum IgG probably indicated an overactive B cell response, which is a distinguishing feature in autoimmune conditions. Several interfering factors are known to mimic the appearance of a monoclonal band on SPE and immunofixation/immunotyping, leading to the false reporting of a monoclonal gammopathy. The presence of cryoglobulins, fibrinogen in plasma samples, or high polyclonal immunoglobulins in CTDs, chronic lymphoproliferative diseases, and chronic active hepatitis are a few of the examples that may falsely present as an M band on protein electrophoresis. Hemolysis may lead to the formation of hemoglobin-haptoglobin complexes as a large band in the alpha-2 region; nephrotic syndrome may present with an increase in alpha-2 and beta bands; and acute phase reactants may lead to an increase in the alpha-1 concentration.

The possibility of monoclonal gammopathy was ruled out in light of the above-mentioned facts and laboratory findings.

A connective tissue disease was the second differential diagnosis. The diagnosis of a new autoimmune disease in a 63-year-old male who was already a diagnosed case of a DCTD, i.e., RA, was challenging due to varied clinical presentation, the presence of multiple autoantibodies on line immunoassay and ANA profile testing, and varied laboratory findings. Late-onset lupus has been defined in the literature by most studies as onset at age ≥ 50 years and is uncommon (found in 12%-18% of SLE). There is less clinical awareness of late-onset lupus, leading to a delay between onset and diagnosis. [4]. RA and SLE have been known to be associated for many years. Due to their distinct genetic backgrounds and pathogenic mechanisms, some have contended that both diseases are intricate and exist as mutually exclusive entities. Nevertheless, contemporary insights are calling into question this perspective, proposing that there is a potential for overlap between the two diseases. Icen et al. explored the frequency of SLE features and their influence on mortality in a cohort of 603 subjects with RA followed over time in a study conducted at the Rochester Epidemiology Project, USA [5]. A cumulative incidence of four SLE features, including arthritis, was found in 15.5% of patients after 25 years of follow-up, which was associated with a twofold increase in the risk of death. “Rhupus” or “rhupus syndrome,” which is inadequately characterized and frequently goes undiagnosed, has features of both rheumatoid arthritis (RA) and systemic lupus erythematosus (SLE) in the same patient, most often sequentially [5].

SLE is a chronic autoimmune disease that is characterized by the presence of multi-systemic manifestations in patients with immunologic abnormalities. While 90% of patients with SLE have joint involvement [1], only a small subset of patients develop erosive arthritis. With the advent of sensitive diagnosing techniques like high-resolution ultrasound with color Doppler and magnetic resonance imaging (MRI), up to 40% of SLE patients with joint involvement are known to develop erosive arthritis. This more aggressive phenotype of SLE has been noted independently and not just in association with rheumatoid arthritis. The term "rhupus" is used to describe the simultaneous presence of erosive symmetrical polyarthritis, a characteristic feature of rheumatoid arthritis (RA), along with clinical indications of systemic lupus erythematosus (SLE), particularly in the presence of anti-double-stranded DNA (anti-dsDNA) and/or anti-Smith antibodies (anti-Sm). Individuals with rhupus exhibit noticeably lower levels of kidney involvement compared to patients diagnosed solely with SLE [6].

In such conditions, establishing a definitive diagnosis can be extremely difficult. Differentials included overlap syndrome (OS) as rhupus, UCTD, MCTD, and late-onset SLE. We discuss the challenges faced in arriving at a final diagnosis in such conditions and the role of line immunoassay in such cases. Polyautoimmunity, a term originally coined by Sheenan and Stanton-King, refers to the coexistence of more than one autoimmune disease in an individual. Substantial evidence supports the idea that autoimmune diseases share varied clinical features, underlying physiological processes, and environmental and genetic triggers. The fact that many ADs share a similar underlying pathology and have a tendency to cluster provides evidence for the participation of common susceptibility genes and similar molecular mechanisms. This suggests a common etiology, which is sometimes referred to as the autoimmune tautology.

In the case described above, active rheumatoid arthritis was ruled out due to the absence of features suggestive of erosive arthritis involving small or large joints, morning stiffness, and the absence of raised values for RF and anti-CCP as per ACR/EULAR criteria 2010. The probability of an OS being “rhupus” was ruled out due to the absence of any erosive deformity. Two differentials that needed to be considered were late-onset SLE and MCTD.

A new connective tissue disease was described by Dr. Sharp and his colleagues in 1972, which was characterized by overlapping features of SLE, systemic sclerosis (SSc), and polymyositis/dermatomyositis (PM/DM), along with the existence of antibodies targeting the U1 small nuclear ribonucleoprotein autoantigen (U1 snRNP) [7]. This condition was designated as mixed connective tissue disease (MCTD) and proposed as a distinct medical condition. However, over the past 40 years, the distinct entity of this disease has been challenged, as the prognosis of the disease is worse than what was originally described by Sharp and colleagues. Moreover, anti-U1sn RNP positivity is not specific, and it may evolve into a DCTD [7]. Three sets of classification criteria for MCTD are currently used: Kasukawa, Alarcón-Segovia, and Sharp, each with varying sensitivity [8-10].

It has been reported in the past that the same patient fulfilled one set of MCTD criteria at the time of diagnosis but not the other. Our patient did not have any symptoms strongly suggestive of an MCTD except for the presence of antibodies against U1 snRNP detected at a dilution of 1:100 by LIA. Moreover, most authors have described MCTD as an independent entity, while some believe that it might represent an early stage of a definite connective tissue disease, e.g., SLE, SSc, or overlap syndrome. Mixed connective tissue disease (MCTD) lacks distinctive clinical features, and there is significant variability in the clinical manifestations among individuals [11].

Prior research has identified several factors that can be used to differentiate between mixed connective tissue disease (MCTD), systemic lupus erythematosus (SLE), and scleroderma. A significant concentration of anti-RNP (ribonucleoprotein) or anti-Smith/RNP (Sm/RNP) antibodies in the blood, in the absence of lupus-specific antibodies like anti-dsDNA or scleroderma-specific antibodies such as anti-Scl-70, is indicative of MCTD. It's important to note that antibodies to dsDNA and Smith antigen are only briefly positive in MCTD. However, if anti-dsDNA or anti-Smith antibodies are constantly dominant, SLE should be strongly considered as the primary diagnosis, since these autoantibodies are highly specific to SLE. In many cases of late-onset arthritis, anti-ds-DNA antibody levels may be low, and manifestations like malar rash, arthritis, and photosensitivity may be less frequent. The absence of certain common complications in SLE, like kidney problems and central nervous system issues, suggests a likelihood of MCTD. Severe arthritis and pulmonary hypertension are frequently observed in MCTD but are not as prevalent in Lupus. Likewise, severe Raynaud's phenomenon and swollen or puffy hands are common features of MCTD but are not typically associated with lupus [12].

## Conclusions

Connective tissue diseases are probably the greatest masqueraders in clinical medicine and present with a wide range of diverse symptoms. Specific classification criteria guide the diagnosis of these disorders. These criteria undergo revisions to incorporate emerging evidence. However, many patients have clinical features and laboratory findings that overlap between these diseases. The patient may also develop new symptoms and signs, and the clinical picture may transform over time. As a result, the clinical features of a patient who presents with an “undifferentiated” connective tissue disease may metamorphose into those of one of the differentiated diseases as described above. Such circumstances make diagnosis very challenging and difficult. This highlights the importance of continued surveillance for newly developing autoimmune diseases, and long-term follow-up is warranted for defining the final phenotype. The possible differential diagnoses in the case described above were monoclonal gammopathy, late-onset SLE, Sjogren syndrome, and MCTD. The final diagnosis was late-onset SLE given clinical features and laboratory findings. Hence, it is always advisable to keep in mind the diagnostic dilemmas that may be faced while encountering chronic inflammatory diseases with overlapping clinical presentations and varied laboratory findings.
